# Different Roles of Mitochondria in Cell Death and Inflammation: Focusing on Mitochondrial Quality Control in Ischemic Stroke and Reperfusion

**DOI:** 10.3390/biomedicines9020169

**Published:** 2021-02-09

**Authors:** Marianna Carinci, Bianca Vezzani, Simone Patergnani, Peter Ludewig, Katrin Lessmann, Tim Magnus, Ilaria Casetta, Maura Pugliatti, Paolo Pinton, Carlotta Giorgi

**Affiliations:** 1 Laboratory for Technologies of Advanced Therapies, Department of Medical Sciences, University of Ferrara, 44121 Ferrara, Italy; marianna.carinci@unife.it (M.C.); bianca.vezzani@unife.it (B.V.); simone.patergnani@unife.it (S.P.); paolo.pinton@unife.it (P.P.); 2 Department of Neurology, University Medical Center Hamburg-Eppendorf, Martinistraße 52, 20251 Hamburg, Germany; p.ludewig@uke.de (P.L.); k.lessmann@uke.de (K.L.); t.magnus@uke.de (T.M.); 3 Department of Neuroscience and Rehabilitation, University of Ferrara, 44121 Ferrara, Italy; ilaria.casetta@unife.it (I.C.); maura.pugliatti@unife.it (M.P.)

**Keywords:** ischemic stroke, ischemic reperfusion, cell death, inflammation, mitophagy, mitochondrial fusion, mitochondrial fission, mitochondrial transfer

## Abstract

Mitochondrial dysfunctions are among the main hallmarks of several brain diseases, including ischemic stroke. An insufficient supply of oxygen and glucose in brain cells, primarily neurons, triggers a cascade of events in which mitochondria are the leading characters. Mitochondrial calcium overload, reactive oxygen species (ROS) overproduction, mitochondrial permeability transition pore (mPTP) opening, and damage-associated molecular pattern (DAMP) release place mitochondria in the center of an intricate series of chance interactions. Depending on the degree to which mitochondria are affected, they promote different pathways, ranging from inflammatory response pathways to cell death pathways. In this review, we will explore the principal mitochondrial molecular mechanisms compromised during ischemic and reperfusion injury, and we will delineate potential neuroprotective strategies targeting mitochondrial dysfunction and mitochondrial homeostasis.

## 1. Introduction

**Figure 1:**
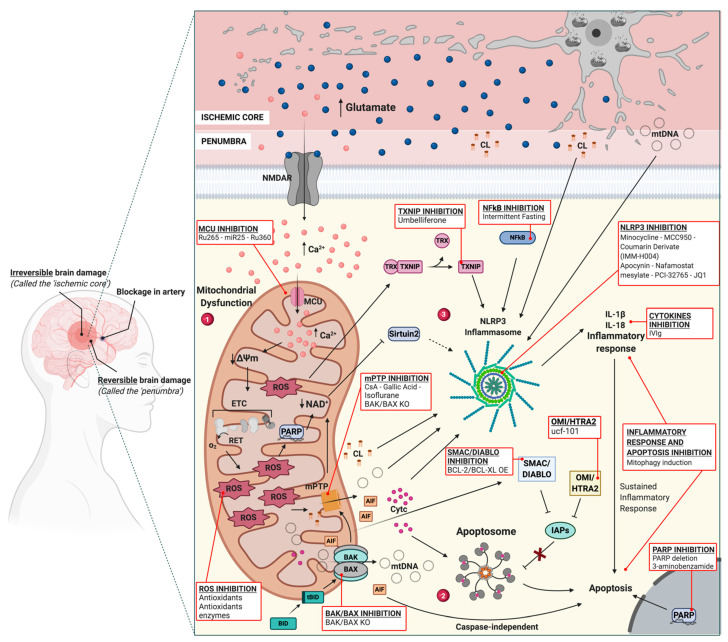
Schematic representation of mitochondrial dysfunctions involved in the ischemic stroke (IS) pathophysiology. The blockage of artery leads to the instauration of two regions, an ischemic core in which necrosis takes over followed by the release of intracellular content, and a penumbra, a reversible damaged area where mitochondrial dysfunctions (1 magenta circle) activate several responses from apoptosis (2 magenta circles) to inflammation (3 magenta circle). Red squares highlight the therapeutic approaches and drugs that improved the ischemic outcome (OE: overexpression; CL: cardiolipin; KO: Knockout). All of the cellular pathways involved in the IS pathophysiology and approaches to overcome mitochondrial damage are widely described in the main text. Created with BioRender.com.

Stroke is one of the leading causes of adult morbidity and mortality in most developing and developed countries. According to the World Health Organization (WHO), each year, 15 million people are affected by stroke worldwide, most of whom experience neurological and physical disabilities or die. The term ischemic stroke (IS) encompasses conditions in which the blood flow to part of the brain is temporarily or permanently reduced, resulting in tissue damage. IS subtypes are categorized based on cause: large-artery atherosclerosis, small-vessel occlusion, cardioembolism, stroke of other determined etiology (e.g., vasculitis), and stroke of undetermined etiology [1]. Middle cerebral artery has been found to be the main affected territory [2]. Oxygen and glucose deprivation (OGD), one of the damaging mechanisms of IS, differentially affects brain regions. Within the first hours from IS onset, the affected brain tissue is mainly divided into two major areas (Figure 1). The first area, named the ischemic core, refers to a cerebral area with a strong decrease in blood flow and oxygen and glucose levels, which results in irreversible damage from a rapid depletion of neuronal energy stores leading. The second area, the ischemic penumbra, surrounds the ischemic core and represents a high-risk region for further damage [3]. This latter area supports the concept of “time is brain” [4]: in fact, in this zone, the blood flow is partially preserved, thus enabling the maintenance of structural integrity and metabolic activity of neuronal cells. However, the ischemic penumbra can become part of the ischemic core if reperfusion is not rapidly restored [5,6], which is the reason why current IS therapies aim to reestablish the vascularization of the ischemic penumbra before irreversible injury occurs. In this regard, endovascular treatment of IS has changed the paradigms of cerebral artery occlusion therapy. Intravenous therapy with recombinant tissue plasminogen activator (tPA) was the only demonstrated therapeutic option, limiting the treatment to a restricted time window of 9 h from the onset of IS [7,8]. Recently published trials have demonstrated the safety and efficacy of endovascular treatment up to 6 h after the onset of IS [9,10,11,12,13,14]. In addition, a new generation of randomized controlled clinical trials with specific selection criteria based on patient characteristics has made it possible to extend the time window in which it is possible to treat patients with IS due to occlusion of proximal anterior circulation vessels with thrombectomy (16–24 h) [15,16,17,18]. However, due to the restricted eligibility criteria [19] for patients to receive these treatments, many stroke patients do not receive active therapies. Although early thrombolysis and revascularization are considered the best treatments for IS, ischemia/reperfusion (I/R) injury (the damage triggered by the rapid restoration of the blood supply to the brain after a period of OGD), antagonizes the benefits of the treatments and exacerbates brain damage [20,21,22]. In light of all these issues, it has become essential to develop effective therapies to counteract the harmful outcomes of IS and I/R by identifying new possible therapeutic targets. In this regard, many works aimed at defining the crucial targetable pathways involved in IS have focused on mitochondrial dysfunction [23,24]. Mitochondria are leading organelles involved in cell homeostasis, a feature that makes them important cell-fate determinants. In addition to be the powerhouses of cells, mitochondria have a crucial role in regulating essential cellular processes ranging from Ca^2+^ signaling, reactive oxygen species (ROS) production, and inflammation to cell death. Following IS, the inadequate oxygen and glucose supply in brain cells results in the alteration of mitochondrial functions, leading to the devastating outcomes of IS and I/R. Thus, targeting altered mitochondrial pathways to recover mitochondrial function represents a promising therapeutic strategy for IS [25]. In this review, we will review the principal mitochondrial molecular mechanisms that function during IS and I/R injury, and we will delineate potential neuroprotective strategies targeting mitochondrial dysfunction and mitochondrial homeostasis.

## 2. From OGD to Cell Death and Inflammation: Targeting Mitochondrial Molecular Mechanisms

### 2.1. Mitochondria, OGD and Ca^2+^ Release in IS

Mitochondria are organelles of bacterial origin considered to be the energy generators of cells. In recent decades, due to their role in regulating a plethora of cellular processes, mitochondria have been identified to play a central role in several brain pathologies, such as Alzheimer’s disease, Parkinson’s disease, traumatic brain injury, IS, and I/R [23,26,27,28,29,30]. Mitochondria are structurally composed of an outer mitochondrial membrane (OMM) enclosing an intermembrane space (IMS). The OMM contains protein channels that ensure membrane permeability to ions, water, and adenosine diphosphate and triphosphate (ADP and ATP, respectively). The inner mitochondrial membrane (IMM) limits the mitochondrial matrix and is considered the reactive center of mitochondrial energy metabolism [31]. In brain cells, mitochondria generate cellular energy by coupling glucose oxidation to ATP production through the electron transport chain (ETC) [32]. Since the last electron acceptor of the ETC for ATP generation is oxygen (O_2_), this process is known as mitochondrial oxidative phosphorylation (OXPHOS). The process includes multienzyme complexes (complexes I–V) embedded in the IMM [33]. The electron carriers NADH and FADH2, which are formed during glycolysis and the Krebs cycle, initiate OXPHOS by donating their electrons to complex I (nicotinamide adenine dinucleotide [NADH]–ubiquinone oxidoreductase) and complex II, respectively. In turn, these complexes transfer electrons to ubiquinol (Q), and then, complex III (cytochrome bc1) moves electrons from Q to cytochrome *c* (Cyt *c*). Complex IV (cytochrome *c* oxidase) transfers electrons from Cyt *c* to molecular O_2_, coupling electron transport with the translocation of protons from the mitochondrial matrix to the IMS. Thus, the proton gradient (ΔpH) that is generated by proton pumps (complexes I, III, and IV) to establish a mitochondrial membrane potential (ΔΨ_m_) is used by complex V (F_1_F_0_-ATP synthase) to catalyze the formation of ATP via the phosphorylation of ADP [33,34,35].

The brain of an adult human being accounts for approximately 2% of the body weight but consumes up to 20% of all the energy the body needs. The functions of the brain depend directly on a sufficient supply of oxygen and energy-rich nutrients. Studies involving the gray matter of rodent brains have shown that even resting neurons consume 13% of the brain energy amount. Most of the consumed energy is used for action potentials and postsynaptic effects of glutamate, which have been predicted to consume 47% and 34% of the energy, respectively [36]. Thus, the energy supplied by mitochondria, representing a considerable fraction of the brain’s total energy, is crucial for ensuring neuronal excitability and survival. Despite being high-energy-consuming cells, neurons contain limited energy reserves [37]; therefore, following IS, OGD affects mitochondrial functions, promoting an extreme reduction in OXPHOS. The major mechanisms mediating brain information processing are all initially powered by oxidative phosphorylation [38]. Failure of mitochondria to compensate for the cell’s request for ATP results in malfunction of the Na^+^/K^+^ ATPase pump [39,40]. Within minutes from IS onset, cells in the ischemic core induce neuronal membrane depolarization and uncontrolled extracellular glutamate release and undergo necrotic cell death [41]. Glutamate is one of the main neurotransmitters of the brain, and binding of glutamate to several types of receptors, such as N-methyl-D-aspartate receptors (NMDARs), a-amino-3-hydroxy-5-methyl-4-isoxazole propionic acid receptors (AMPARs), and kainate receptors (KRs) [42], is crucial for a wide variety of neuronal functions [43]. These receptors are not exclusively expressed by neurons but also by glial cells. Thus, the excitotoxicity promoted by glutamate release involves several brain cell subpopulations [44,45,46]. Under IS conditions, due to the high levels of extracellular glutamate, cells in the penumbra are subjected to hyperactivation of glutamate receptors, promoting a substantial influx of Ca^2+^ into the cells [47,48,49]. Ca^2+^ ions, as second messengers, have been associated with cell death and survival [50,51,52] and are implicated in several pathologies [53,54]. Mitochondria are strictly connected to Ca^2+^ signaling and are key actors in buffering cytosolic Ca^2+^ levels [54,55,56]. Several channels and uniporters have been identified to control calcium influx and efflux in mitochondria [57]. The highly selective mitochondrial calcium uniporter (MCU) located in the IMM is a crucial player in maintaining intracellular Ca^2+^ homeostasis and promotes the uptake of Ca^2+^ from the cell cytosol into the mitochondrial matrix [58]. Following IS, an increase in the mitochondrial Ca^2+^ concentration triggers a cascade of events ranging from mitochondrial permeability transition pore (mPTP) opening [59,60] and dissipation of ΔΨ_m_ to an increase in ROS production, leading to inflammation and eventually cell death [50,61,62,63,64] (Figure 1). In vitro studies have indicated that downregulation of MCU by the microRNA miR-25 protects cardiomyocytes against oxidative damage [65]. In another study, intraperitoneal injection of Ru265, a cell-permeable MCU inhibitor, suppressed ischemia-induced sensorimotor deficits and brain injury [66]. These findings might suggest therapeutic targets for oxidative stress-related diseases such as IS.

### 2.2. Mitochondria and Cell Death in IS

Neuronal death in IS is strongly associated with the modulatory mechanisms of mitochondria. Consequently, mitochondrial dysfunction has become an attractive target for neuroprotection against ischemic injury [7]. Necrosis and apoptosis pathways have been associated with both the ischemic core and penumbra [67]. However, it is widely accepted that mitochondrial dysfunction in the ischemic core predominantly leads to necrotic cell death due to a bioenergetic catastrophe resulting from ATP depletion in the first minutes post stroke. In the penumbra, where low ATP levels are maintained and cells are impaired but metabolically active, apoptosis is the main death pathway, and apoptosis takes place later, for hours or days [68,69,70]. In this regard, it has been reported that after reperfusion, marked caspase activation is present in the penumbra but not in the core [71]. Proapoptotic proteins belonging to the B cell lymphoma (BCL-2) family, such as BID, BAK and BAX [72,73,74], are heavily upregulated during reperfusion injury. Following IS, increased mitochondrial Ca^2+^ leads to BID cleavage into truncated BID (tBID) [75]. At the mitochondrial membrane, tBID interacts with proapoptotic proteins such as BAK and BAX. Activated BAD translocates to the OMM, resulting in the inhibition of the survival protein BCL-2, inducing BAX to open the mPTP and thus causing the release of Cyt *c* and the consequent formation of the apoptosome [74,76,77] (Figure 1). Of note, loss of the proapoptotic proteins BAK and BAX has been shown to reduce mitochondrial membrane permeability, resulting in resistance to mitochondrial calcium overload and necrotic cell death [74]. In addition, reductions in stroke volume and apoptosis were demonstrated in BID-deficient mice following middle cerebral artery occlusion (MCAO) [78].

The opening of the mPTP in the IMM allows the release of a variety of solutes into the cytosol, among which apoptosis-inducing factor (AIF) and mitochondrial Cyt *c* have been implicated in neuronal cell death after IS [79,80,81,82,83]. Targeting mPTP opening represents a promising strategy for stroke therapy. Mice that are deficient in the mPTP component cyclophilin D (CypD) have reduced infarct size after MCAO [84]. Consequently, administration of the chemical CypD inhibitors cyclosporine A (CsA) and gallic acid has been demonstrated to significantly diminish infarct size in vivo [85,86,87]. Additionally, isoflurane preconditioning has been reported to inhibit mPTP opening and elicit ischemic brain tolerance by activating cannabinoid receptor 1 (CB1R) [88]. The basic structure of the mPTP remains open to debate [89,90]; thus, further studies should be conducted to understand the molecular composition of the mPTP and to develop treatments targeting mPTP opening [90].

As a consequence of mPTP opening, AIF is released and moves to the nucleus, causing DNA fragmentation and cell death through a caspase-independent pathway [91,92].

Once in the cytosol, Cyt *c* binds to apoptotic protease activating factor 1 (APAF-1) and procaspase-9 to form the apoptosome. In turn, the apoptosome activates caspase-9, and the consequent activation of caspase-3 promotes the cleavage of nuclear DNA repair enzymes, such as poly (ADP-ribose) polymerase (PARP). PARP cleavage into 89 and 28 kDa products [93] leads to nuclear DNA damage and apoptotic cell death. In vivo studies have reported that PARP genetic disruption [94] and PARP inhibition [95] protect against ischemic damage, showing a decreased infarct volume (Figure 1).

In addition to Cyt *c*, two other proteins released from the IMS promote caspase-dependent apoptosis: second mitochondria-derived activator of caspase/direct IAP-binding protein of low pI (Smac/DIABLO) and Omi stress-regulated endoprotease/high-temperature requirement protein A2 (Omi/HtrA2) [96]. These proteins bind and block the action of inhibitor-of-apoptosis protein (IAP) family members, which inhibit caspase-3, caspase-7, and caspase-9, thus preventing IAP function and promoting caspase activity and cell death [97]. In vivo studies on MCAO-induced IS have shown the overexpression of mitochondrial proapoptotic proteins such as Smac/DIABLO and HtrA2/Omi following IS [98,99]. Of note, a reduction in DNA damage in the post-ischemic tissue has been shown in rats treated with inhibitors of Omi/HtrA2 activity, such as ucf-101, before induction of temporary ischemia [98]. In addition, X-linked inhibitor of apoptosis (XIAP), a member of the IAP family, is inhibited in MCAO rat cortices via the upregulation of allograft inflammatory factor 1 (AIF) and XIAP-associated factor 1 (XAF1), which act as antagonists of apoptosis inhibitors in the penumbra [100,101]. Interestingly, BCL-2 and BCL-XL inhibit the release of Smac/DIABLO, resulting in the activation of XIAP [102,103,104] (Figure 1). It has also been reported that BCL-2 transfection in the penumbra is able to block the nuclear translocation of AIF, improving cortical neuron survival [105]. Furthermore, inhibition of caspase-3 in a rat MCAO model inhibited apoptosis, resulting in a protective effect on brain tissue [106]. However, it has been demonstrated that caspase inhibition protects against focal but not global ischemia [107].

### 2.3. Mitochondria and ROS Production in IS

Although IS per se leads to an impairment of mitochondrial function with devastating effects, I/R exacerbates ischemic damage. Upon I/R, oxygen and glucose levels are quickly reestablished, resulting in overshooting activity of the mitochondrial ETC, which results in a burst of ROS levels that worsens the ischemic outcome [108]. Mitochondria represent an essential source of ROS, making them important players determining the severity of reperfusion damage [109,110]. Under physiological conditions, mitochondrial ROS are mainly generated from complexes I and III [111]: approximately 2% of the total electrons transported across the mitochondrial respiratory chain react with molecular oxygen, generating a metabolic byproduct of respiration, the superoxide anion O_2_^−^ [112,113,114]. Upon I/R, complex I has been assessed as the major source of ROS. Following I/R, oxidized succinate, markedly increased during ischemia, provides the initiating burst of ROS production at complex I via reverse electron transport (RET) [115,116] (Figure 1). Under physiological conditions, ROS are important second messengers involved in several cellular pathways [117], and adequate antioxidant systems limit damage to cellular constituents. However, in pathological conditions such as IS and I/R, the balance between oxidant and antioxidant molecules is destroyed, resulting in the generation of oxidative stress conditions. In addition to O_2_^−^, ROS include hydrogen peroxide (H_2_O_2_) and hydroxyl radicals (OH^·^), all of which have intrinsic properties enabling them to react with different biological targets ranging from proteins, carbohydrates, and lipids to nucleic acids [118,119]. Thus, ROS-induced brain changes after cerebral stroke, including macromolecular damage and activation of intracellular signaling pathways, worsen outcome [120,121]. Diverse approaches targeting ROS upstream and downstream have been tested to ameliorate stroke outcome. For example, injection or oral administration of the antioxidant vitamin E and alpha-lipoic acid has been shown to decrease infarct size after experimental stroke [122,123]. Moreover, patients showed increased antioxidant capacity and reduced lipid peroxidation products after supplementation with antioxidant vitamins within 12 h of the onset of acute IS. In contrast, high-dose vitamin E supplements have been suggested to increase all-cause mortality [124,125]. Administration of dehydroascorbic acid (DHA), a blood–brain barrier transportable form of vitamin C, and EPC-K1, a phosphate diester of vitamins C and E, ameliorated outcomes in a mouse MCAO model, while preclinical administration did not show a neuroprotective effect [126,127,128]. Apart from antioxidant vitamins, other studies based on free radical scavengers such as edaravone, N-acetylcysteine, and NXY-059 have revealed improved neurological outcomes in rodent and primate models, but the performance of exogenous antioxidants in recent clinical trials has been inconsistent [129,130,131,132].

### 2.4. Mitochondria and Inflammation during I/R

Over the years, greater attention has been given to the role of inflammation in IS. Although immune responses are designed to limit damage, it has been demonstrated that sustained neuroinflammation contributes to cell death and injury after stroke. The priming of the neuroinflammatory process mainly occurs in the penumbra area and is attributable to the release of cellular contents and proinflammatory molecules from necrotic cells in the ischemic core. These inflammatory triggers result in increased mitochondrial Ca^2+^ uptake, mitochondrial ROS overproduction, and mPTP opening, leading to sustained inflammation (Figure 1). In particular, mitochondrial dysfunction is associated with nucleotide-binding domain and leucine-rich repeat-containing protein 3 (NLRP3) inflammasome activation [133,134,135]. This inflammasome is characterized by the cytosolic receptor NLRP3 and the adaptor protein apoptosis-associated speck-like protein containing CARD (ASC), which binds pro-caspase-1. Pro-caspase-1 self-catalyzes into its active form, caspase-1, and cleaves both pro-IL-1 and pro-IL-18 into active proinflammatory cytokines, which are then released into the extracellular environment [136]. All NLRP3 components have been found to be upregulated in the first hours and days from stroke onset [137,138]. In addition, inhibition of NLRP3-mediated neuroinflammation has been associated with the improvement of IS outcome, suggesting the NLRP3 inflammasome as a promising target for treating IS [40,139,140,141]. As reported above, mitochondrial dysfunction contributes to the activation of the NLRP3 inflammasome in several ways: mitochondrial NAD^+^ decrease, mitochondrial ROS overproduction, BAK/BAX macropore formation, mPTP opening, and damage-associated molecular pattern (DAMP) release [142] (Figure 1).

Loss of mitochondrial NAD^+^ following ischemic insult mainly results from mPTP activation, which promotes the depletion of matrix NAD^+^ [143], and from activation of mitochondrial PARP, which degrades intramitochondrial NAD^+^ [144,145]. The diminished concentration of the coenzyme NAD^+^ inactivates Sirtuin 2, an NAD-dependent α-TUBULIN deacetylase, resulting in accumulation of acetylated α-TUBULIN. Acetylated α-tubulin mediates DYNEIN-dependent NLRP3 assembly. Thus, reduced NAD^+^ levels contribute to NLRP3 inflammasome activation [146] (Figure 1).

Another triggering event for NLRP3 activation is ROS overproduction [134,147]. Although ROS have been shown to directly regulate the NLRP3 inflammasome [134], they are also required to promote the transcriptional activation of NFκB [148], which promotes NLRP3 inflammasome activation in neurons following IS [149,150]. In this respect, intermittent fasting (IF) has been reported to decrease the activation of the NF-κB and MAPK signaling pathways, attenuating inflammasome activity in IS [151]. Furthermore, oxidative stress regulates the activation of NLRP3 by promoting thioredoxin (TRX)-interacting protein (TXNIP). In fact, in response to mitochondrial ROS release, TXNIP dissociates from TRX and translocates to the mitochondria-associated membrane (MAM), where it binds to NLRP3, inducing its activation and resulting in maturation and secretion of IL-1 and IL-18 [134]. It was shown that curcumin reduced TXNIP expression and inhibited NLRP3 inflammasome activation in the hippocampus under the condition of glutamate neurotoxicity [152]. Pretreatment with umbelliferone (UMB), a natural antioxidant belonging to coumarin derivatives, for 7 consecutive days ameliorated neurological outcomes, infarct volume, and brain edema after MCAO in rats through the inhibition of the TXNIP/NLRP3 inflammasome [153]. Thus, targeting TXNIP could protect the brain against ischemic injury due to its role in activating the NLRP3 inflammasome [134,154,155,156] (Figure 1).

Following IS, BAK, and BAX have been shown to form stress-induced large macropores associated with the outflow of matrix components, including the mitochondrial genome [157]. As stated before, the opening of the mPTP allows the release of mitochondrial material, such as mitochondrial DNA (mtDNA), ATP, Cyt *c*, and mitochondrial lipids, into the extracellular space [158]. Once released, these proinflammatory signals are recognized by cells of the innate immune system, provoking a local or systemic response [159]. Interestingly, current evidence indicates that DAMP release has prognostic value in human diseases [160], which could be mainly due to the involvement of DAMPs in NLRP3 inflammasome activation. Among the different DAMPs, cardiolipin (CL) and oxidized mtDNA are well-known activators of NLRP3 [161,162] (Figure 1).

Since NLRP3 is required for mtDNA release, a positive feedback loop between NLRP3 activation and mtDNA release could exist; indeed, autophagy proteins have been found to inhibit NLRP3 inflammasome-mediated mtDNA release [163]. Fann and colleagues have provided evidence that intravenous immunoglobulin (IVIg) therapy, used for various inflammatory and autoimmune diseases, exerts protective effects on neurons during ischemic conditions by modulating NLRP3 and NLRP1 inflammasome levels, therefore downregulating the proinflammatory cytokines interleukin (IL)-1β and IL-18 [138,164]. Moreover, interferon β, which can repress NLRP1 and NLRP3 inflammasome activity, has been shown to reduce infarct size and neurological deficits after MCAO [165,166]. Several studies have highlighted the neuroprotective effect of minocycline, a tetracycline antibiotic, in focal cerebral ischemia injury animal models, showing that early treatment or pretreatment prevents the activation of microglia and attenuates NLRP3 inflammasome signaling [167,168,169,170]. Accordingly, minocycline administration in IS patients resulted in improved neurological function [171,172]. Another interesting compound that affects neuroinflammation is the coumarin derivative IMM-H004, which has been shown to target chemokine-like factor 1 and suppress the activation of the NLRP3 inflammasome in permanent (p)MCAO in vivo models [173]. Supporting this mechanism, the best characterized selective NLRP3 inflammasome inhibitor, MCC950, has been shown to provide protection in mouse transient (t)MCAO by reducing IL-1β levels [139,174,175,176]. In the same way, several other inhibitor molecules, such as apocynin, nafamostat mesylate, PCI-32765, and JQ1, had beneficial effects on IS outcome, reducing NLRP3 inflammasome activity [177,178,179,180], suggesting that targeting the NLRP3 inflammasome is a promising therapeutic approach for IS treatment [181].

## 3. Mito-Recovery: Regaining Mitochondrial Homeostasis in IS and I/R

Mitochondria are highly dynamic organelles that unceasingly fuse and divide thanks to the coordinated activity of mitochondrial fission and fusion, mitochondrial biogenesis, and mitophagy. All these activities permit control of the number, morphology, quality, and distribution of mitochondria in cells. These mitochondrial dynamics are essential for regulating diverse mitochondrial functions, such as metabolism, inflammation, cell death mechanisms, energy production, and cell differentiation and movement [182].

### 3.1. Mitochondrial Biogenesis and IS

**Figure 2:**
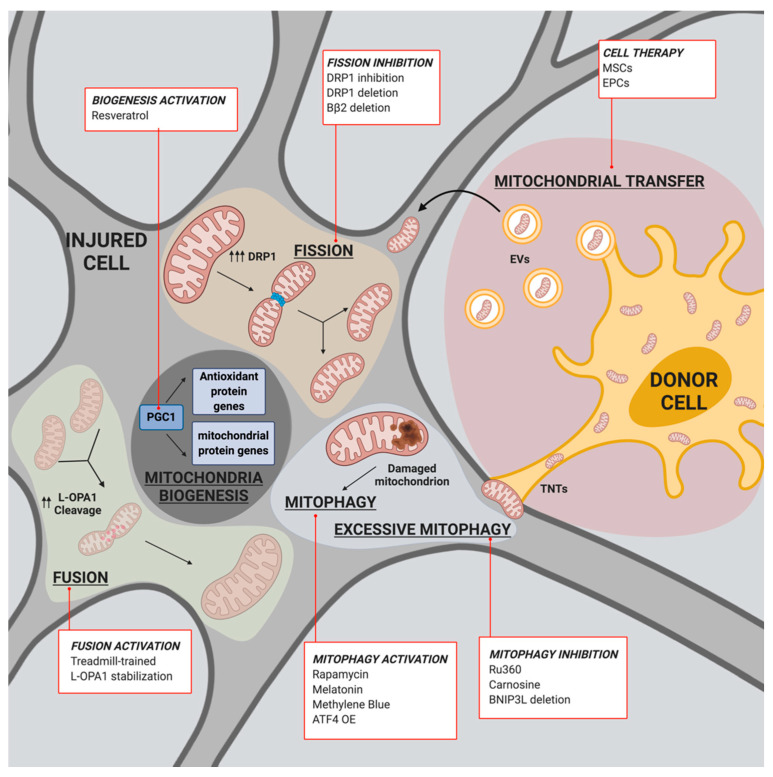
Mito-recovery in IS. Schematic representation of the mitochondrial mechanisms involved in the mito-recovery process during IS. In the red encircled boxes are indicated the therapeutic approaches and/or therapeutic drugs that affects each of the represented pathway (OE: overexpression). Created with BioRender.com (accessed on 8 February 2021).

Mitochondrial biogenesis is the cellular process by which pre-existing mitochondria grow or divide to generate new ones. Since it requires the synthesis, import and incorporation of approximately 1500 proteins (the majority of which are encoded by the nuclear genome) and a large amount of lipids (to create mitochondrial membranes) and the replication of mtDNA, mitochondrial biogenesis is the result of a highly interconnected pathway. It is clear that mitochondrial biogenesis necessitates the harmonized transcription of mitochondrial and nuclear genes. This coordination is permitted by a multistep process in which the peroxisome proliferator-activated receptor-γ coactivator (PGC)-1 protein family plays a central role [183]. Once activated by phosphorylation or deacetylation, PGC-1α, the main regulator of mitochondrial biogenesis, stimulates the expression and activation of a series of nuclear factors, such as nuclear respiratory factor (NRF)-1 and NRF-2 (which are responsible for regulating nuclear-encoded mitochondrial genes involved in the ETC), and nucleus-encoded mitochondrial proteins, including mitochondrial transcription factor A (TFAM) (which is necessary for initiating the transcription and duplication of mtDNA) [184]. Mitochondrial biogenesis is regulated by physiological signals activated during particular conditions, such as endurance exercise or caloric restriction, or even pathological conditions, such as cancer, neurodegeneration and IS [185,186,187,188]. Consistent with this, in a rat model of hypoxic/ischemic brain injury, it has been observed that increased activity of PGC-1α correlates with an increase in mitochondrial number, mtDNA amount, and mitochondrial protein expression, leading to neuroprotective effects [189]. Similarly, increased PGC-1α levels protected neural cell cultures from oxidative stressor-mediated death [190]. Additionally, in vivo, hippocampal neurons exposed to transient global ischemia showed upregulation of PGC-1α with consequent enhanced mitochondrial biogenesis and upregulation of mitochondrial ROS-detoxifying enzymes, such as superoxide dismutase 2 (SOD2) and uncoupling protein 2 (UCP2), suggesting that PGC-1α activation also protects neurons from oxidative stress [191]. Accordingly, PGC-1α-null mice display an increased sensitivity to oxidative stress [192]. Overall, these findings suggest that mitochondrial biogenesis represents a protective mechanism by which cells engage to counteract ischemic conditions and could be considered a putative target for novel strategies aimed at reducing mitochondrial impairment in stroke. In this regard, it has been found that resveratrol is able to modulate mitochondrial biogenesis. This potent ROS scavenger has a protective role against ischemic injury and is capable of activating Sirtuin 1 (SIRT1), which in turn catalyzes PGC-1α deacetylation, finally leading to mitochondrial biogenesis [193] (Figure 2).

### 3.2. Mitochondrial Fusion/Fission and IS

In addition to mitochondrial biogenesis, mitochondrial fusion is also a finely controlled mechanism. In fact, it is primarily controlled by three dynamin-related proteins, namely, mitofusin (MFN) 1 and 2, and optic atrophy 1 (OPA1), which regulate the fusion process via the OMM and the IMM, respectively [194,195]. By hydrolyzing GTP, these proteins permit two neighboring mitochondria to join, becoming one mitochondrion and sharing proteins, DNA and metabolites. The fusion of the OMM is mediated by MFNs, which allow a single mitochondrion to tether to another mitochondrion, creating either homodimers between MFN2 molecules or heterodimers between MFN1 and MFN2 molecules. Consistently, genetic ablation of MFN1 and MFN2 results in a lack of mitochondrial fusion and embryonic lethality, while further mutations in MFNs cause the autosomal dominant neurodegenerative disease Charcot-Marie-Tooth type 2A [196]. On the other hand, OPA1 is responsible for the fusion of the IMM. OPA1 is ubiquitously expressed and exists in long (L-OPA1) and short (S-OPA1) forms as a result of proteolytic cleavage by IMS proteases, such as presenilin-associated rhomboid-like protease (PARL), overlapping activity with m-AAA protease (OMA1) and matrix- and intermembrane space ATPases (the m-AAA and i-AAA proteases, respectively), which are associated with a number of cellular activities [197]. Functionally, OPA1 is translated in the cytosol and then imported into the mitochondria, where a series of peptidases remove the mitochondrial import sequence to generate the L-OPA1 isoform. Once anchored to the IMM, L-OPA1 is further processed and cleaved by other IMS proteases, generating the S-OPA1 form. It has been demonstrated that these cleavage steps are required for correct IMM fusion [197]. Although both L-OPA1 and S-OPA1 are required to control fusion of the IMM, it has been reported that under stress conditions, the L-OPA1 isoform alone may be able to trigger mitochondrial fusion [198]. Interestingly, in the tMCAO model, L-OPA1 is excessively cleaved [199] (Figure 2). Consistently, local overexpression of OPA1 isoform 1 with a defective S1 cleavage site was able to restore mitochondrial structures as well as motor functions after the induction of cerebral I/R injury [199]. Interestingly, treadmill-trained rats showed significantly increased expression of OPA1 and displayed reduced brain edema compared to rats in the untrained group upon tMCAO, supporting the idea that exercise training may be a neuroprotective strategy during brain ischemic injury [200,201].

Overall, these data suggest that mitochondrial fusion plays a key role in recovery from IS injury. Accordingly, the downregulation of MFN2 aggravates I/R injury in vitro [202], and a novel MFN2 missense mutation (c.1367C → T) has been associated with early-onset stroke in humans [203].

As reported, mitochondrial fusion describes the process by which individual mitochondria join to become one. In contrast, the mitochondrial fission process refers to the division or fragmentation of a single mitochondrion into two mitochondria. The first step, and the starting point, of this process is the replication of mtDNA in the matrix, which ensures that the mitochondrial genome will pass to new mitochondria. The second step of the mitochondrial fission machinery is the recruitment of the fission protein dynamin-related protein 1 (DRP1) from the cytosol to the OMM [204]. Here, DRP1 interacts with other key mitochondrial proteins involved in mitochondrial scission: human fission factor-1 (FIS1), mitochondrial fission factor (MFF), and mitochondrial dynamics proteins (MIDs) 49 and 51 [205]. After binding with these proteins, DRP1 moves to future scission sites on the OMM and oligomerizes into a helical structure, forming a collar that encircles and constricts the mitochondria, leading to ultimate disruption of the membranes. After division, DRP1 returns to its monomeric structure and returns back to the cytosol. DRP1 oligomerization can be triggered by different signals that lead to the phosphorylation of its two activating sites. One site may be phosphorylated by cyclin-dependent kinase 1 (CDK1), ERK1/2, and PKCδ, and it is named the upstream phosphorylation site (SerCDK1). The second site (SerPKA) can be activated by cyclic AMP-dependent protein kinase (PKA), Rho-associated coiled coil-containing protein kinase 1 (ROCK1) and calcium/calmodulin-dependent protein kinase Iα (CaMKIα) [206,207,208]. Another division signal is executed by CL, which can interact with DRP1 to stimulate its oligomerization [209]. It is widely recognized that mitochondrial fission has an important role in human diseases; consistently, it has been found that DRP1 is dysregulated in different tumors, such as breast, lung, pancreatic, and brain cancers [210,211]. Regarding the central nervous system (CNS), posttranslational modifications of DRP1 have been observed to cause excessive mitochondrial fragmentation and neuronal loss in Alzheimer’s disease [212]. In in vitro models of glutamate toxicity and oxygen and glucose deprivation (OGD) and in in vivo tMCAO models, it has been observed that inhibition of DRP1 has a neuroprotective effect [213]. Consistently, another work reported that the administration of a small molecule inhibitor of DRP1, Mdivi-1, both in vitro in SH-SY-5Y cells and in vivo in tMCAO mice ameliorated the outcome of ischemic-induced mitochondrial injury [214]. The effective action of Mdivi-1 has been highlighted in a recent study reporting that Mdivi-1 is not a specific Drp1 inhibitor but acts by reversibly inhibiting complex I, thus modifying mitochondrial ROS production [215]. However, inhibiting DRP1 remains an ongoing challenge. Indeed, it has been found that the deletion of a neuron-specific DRP1 activator, Bβ2 (a mitochondria-localized protein phosphatase 2A regulatory subunit), in vivo ameliorated excessive stroke damage [216]. Interestingly, DRP1 also participates in processes other than mitochondrial fission in vascular tissues following ischemic injury, such as ischemia-induced autophagy, apoptosis, and metabolic pathways [217].

Further supporting the idea that fusion and fission mechanisms play an important role in delineating the extent of injury in IS, in the ischemic penumbra, both DRP1 and OPA1 were found to be upregulated at 2 days after tMCAO (with a peak at 14 days for DRP1), indicating that mitochondrial fission proceeds for days [218] (Figure 2). Of note, using a transgenic mouse model expressing a fluorescent protein fused to mitochondria in brain cell subpopulations, it has been demonstrated that following ischemic insult, mitochondrial dynamics are heterogeneous among neurons and astrocytes and between different hippocampal areas. Though the timing varies, cells that survive IS are able to shift their mitochondrial dynamics from fission to fusion in order to regain their tubular shape. Higher fusion events coincide with the transient elevation of MFN2 and OPA1 levels and most likely constitute an attempt to initiate fusion of fragmented mitochondria in brain cells [219]. In agreement with this, a recent work suggests that the mitochondrial damage and fragmentation provoked by mild and moderate ischemic injury can be reverted [220], indicating that mitochondrial dynamics are a potential target for therapeutic interventions in IS.

### 3.3. Mitophagy and IS

Cells have evolved specific mechanisms of quality control to preserve the right mitochondrial population. Among them, the first contributor is sequestration of injured mitochondria by a specific autophagy process named mitophagy [221]. During the mitophagic process, damaged and aged mitochondria are recognized by the autophagic machinery and then degraded. Different mechanisms regulate this recognition and the subsequent catabolic process. Among them, the first to be discovered was observed in reticulocytes during the differentiative process, which was characterized by a prominent loss of mitochondria that was promoted by NIP3-like protein X (NIX/BNIP3L) [222]. In the last decade, several other mitophagic regulators have been unveiled, such as the OMM proteins FUNDC1, FK506-binding protein 8 (FKBP8), and BCL2L13 [223,224,225]. However, undoubtedly, the best characterized molecular alliance regulating mitophagy is the PTEN-induced kinase 1 (PINK1)/Parkin axis [226]. Under normal conditions, PINK1 is continuously imported into mitochondria, where it is subjected to proteolytic cleavage and then exported back to the cytosol for its degradation. Upon a stress signal, PINK1 accumulates at the OMM, where it autophosphorylates and dimerizes. In this active state, PINK1 can recruit and activate the E3 ligase Parkin to form an active phospho-ubiquitin (Ub)-dependent enzyme [227,228]. Parkin can then promote the ubiquitination of several OMM proteins [229], which provides signals for the Ub-binding autophagy receptors p62/sequestome, NDP52, optineurin (OPTN), and NBR1, to connect the damaged mitochondria to phagosomes for clearance [230,231]. Supporting the idea that mitophagy is critical for conserving an appropriate mitochondrial population essential for cell survival, mitophagic dysfunctions are involved in the development of several human pathologies, such as neurodegenerative disorders [232,233], cancer [234], cardiovascular diseases [235], and cerebral ischemia [236]. In cerebral ischemia, the effective role of mitophagy remains controversial. In fact, the logical assumption would be that mitophagy exerts a protective role, since it functions to remove damaged mitochondria after ischemic attack. In support of this idea, it has been demonstrated that mitophagy-related mitochondrial clearance is activated during cerebral I/R both in vivo and in vitro. Accordingly, administration of an autophagic inducer, rapamycin, in tMCAO rats activates mitophagy, resulting in the attenuation of mitochondrial dysfunction and improved neurological outcomes [237]. Following cerebral ischemia, treatment with different compounds, such as melatonin, methylene blue, and rapamycin, promotes the inhibition of ROS generation and NLRP3 inflammasome activation by enhancing the mitophagy process [237,238,239] (Figure 2). The overexpression of activating transcription factor 4 (ATF4) has been shown to improve cerebral I/R injury by reducing NLRP3 inflammasome activation through parkin-dependent mitophagy [240]. As further evidence in support of the fundamental role of mitophagy in counteracting neuroinflammation and thus IS injury, autophagy proteins are able to inhibit the release of mtDNA mediated by the NLRP3 inflammasome [163].

However, mitophagy may also have a negative effect in the context of stroke (Figure 2). In fact, it may provoke uncontrolled degradation of mitochondria that can result in cell death. Consistent with this, during the reperfusion phase, mitochondria are subjected to a loss of mitochondrial membrane potential that may be sufficient to activate mitophagy [241]. Although BNIP3L knockout mice show impaired mitophagy and aggravated cerebral I/R injury [242], BNIP3 is able to promote excessive mitophagy, and as such, BNIP3 silencing has been associated with neuroprotection and rescues neurons from ischemia/hypoxia [243]. Interestingly, the MCU inhibitor Ru360 inhibits excessive mitophagy and protects neurocytes from I/R injury [244]. In addition, it has been observed that the endogenous pleiotropic dipeptide carnosine exerts neuroprotective activity against ischemic brain damage by attenuating deleterious mitophagic processes [245]. Therefore, a greater understanding of the precise molecular processes and timing underlying the involvement of mitochondria homeostasis pathways in IS and I/R is essential for developing neuroprotective strategies for treating cerebral ischemic injury.

### 3.4. Mitochondrial Transfer and IS

As described above, mitochondrial homeostasis plays a crucial role in maintaining the functionality of the CNS. I/R injury broadly affects this finely tuned balance, leading to mitochondrial disruption and neuronal cell loss. All the mitochondrial processes previously discussed imply that each cell is responsible for the regulation (i.e., production and fission-fusion mechanisms) and degradation (i.e., mitophagy) of its own mitochondria. This assumption was correct until 2004, when Rustom and colleagues performed three-dimensional (3D) live-cell microscopy on cultured cells and identified a novel cell-to-cell communication network. In this study, they distinguished nanotubular structures, named tunneling nanotubes (TNTs), involved in the transfer of membrane vesicles and organelles [246]. Further studies showed that TNTs work bidirectionally, indicating that these nanotubes are similar to intracellular highways involved in cell communication [247,248,249]. Mitochondria, as separate organelles, take part in this intracellular exchange, and TNTs represent only one of the possible exchange routes. In fact, mitochondrial transfer also occurs via membranous extracellular vesicles (EVs), such as exosomes (30–150 nm) and macrovesicles (30–1000 nm) [250,251], and through gap junctions, cell fusion and direct uptake [252,253,254]. Currently, it is widely recognized that mitochondrial intracellular transfer is not restricted to a specific cell type but occurs in vivo and in vitro between different cellular subsets, including lymphocytes, neurons, and cardiomyocytes, and it is present in different pathophysiological conditions [247,254,255,256,257,258]. Notably, it has been demonstrated that neurons are able to dispose of and recycle damaged mitochondria by releasing and transferring them to astrocytes [255]. This ability was first observed in mouse retinal ganglion cell axons, which extrude damaged mitochondria by generating protrusions in direct contact with adjacent astrocytes; after transfer, the mitochondria are degraded through a process named transmitophagy [255]. Since intracellular communication works bidirectionally, it has been shown that, together with the transmitophagic process, astrocytes are responsible for transferring healthy mitochondria to ischemic neurons in vivo following transient focal cerebral ischemia via CD38/cyclic ADP ribose-mediated mechanisms [256]. CD38, a catalyzer of the synthesis of cyclic ADP ribose in mitochondrial membranes, has a prominent role in vehiculating mitochondrial transfer [259]. Hence, CD38 signaling suppression results in reduced mitochondrial exchange and worsens the neurological outcome in the transient focal cerebral ischemia mouse model [256]. With this knowledge, the understanding of the neuroglia-protective role of astrocytes assumes new facets: healthy astrocytic mitochondria may play an essential role in preventing neuronal damage by reducing oxidative stress and excitotoxicity and by replacing the damaged neuronal mitochondrial pool [30,256,260]. Accordingly, CD38 in the CNS is mainly expressed by glial cells, and its upregulation in astrocytes is induced by neurons via glutamate release [261]. However, even if this natural host repair mechanism is unable to overcome all the damage induced by I/R injury during stroke, it provides a fundamental basis for a cell therapy-based approach. Since mitochondrial transfer occurs via both EVs and TNTs, therapeutic approaches have developed in two distinct ways. In the first case, stem cells serve as producers of EVs containing functional mitochondria that are then collected and dispensed to the affected recipient. Following this approach, functional xenogenic mitochondria administered to ischemic rat brains through local intracerebral or systemic intra-arterial injection significantly attenuate the brain infarct area and neuronal cell death, restoring motor performance [262]. Similarly, autologous transplantation of skeletal muscle-derived mitochondria into the lateral ventricles of ischemic rats resulted in a reduction in brain infarct volume and in the reversion of neurological deficits [263]. Moreover, infused mitochondria diffused to the boundary and ischemic penumbra areas, which decreased cellular oxidative stress and apoptosis, attenuated reactive astrogliosis and promoted neurogenesis [263]. In the second scenario, autologous or heterologous stem cells are directly transplanted into the ischemic brain, where they help reduce the neuroinflammatory process and promote the restoration of CNS homeostasis. Due to their low immunogenicity and neuroprotective potency, mesenchymal stem cells (MSCs) represent one of the most promising candidates for IS cytotherapy [264,265]. MSCs can be isolated from virtually all adult tissues and are endowed with mesodermal differentiation capabilities, secretory capacity and the ability to promote tissue regeneration [266]. Several studies performed on IS animal models revealed that the administration of human bone marrow-derived MSCs (bmMSCs) after stroke reduces the infarct area dimensions, enhances angiogenesis and ameliorates functional deficits, thus improving the recovery process [267,268,269,270,271], even though the mechanisms underlying this recovery are still debated. Evidence suggesting that the therapeutic effect of bmMSC transplantation includes not only studies showing the ability of bmMSCs to differentiate into neuronal cells [272] but also recent studies highlighting the central role of mitochondrial transfer in improving IS outcome after cell administration [273,274,275]. Indeed, it has been reported that bmMSCs are able to transfer functional mitochondria to ischemic endothelial cells via TNTs in vitro, rescuing aerobic respiration and decreasing the apoptotic rate [275,276,277]. Moreover, bmMSCs are also able to exchange intracellular contents with cortical neurons once cocultured in vitro [278]. Although the molecular mechanism beyond this transfer remains to be fully identified, Miro1, a key participant in mitochondrial trafficking, is involved in promoting mitochondrial transfer between cells [279]. Accordingly, bmMSCs express higher levels of Miro1 upon cocultivation with neurons, which correlates with an increased neuroprotective effect, indicating that neural cell-to-cell interactions improve bmMSC neuroprotective abilities [278]. In the same way, decreasing Miro1 expression in bmMSCs resulted in a reduced metabolic benefit in cocultured neurons [280]. A recent study showed that different proinflammatory and mitochondrial motility genes, including Miro1 and TNFAIP2, were upregulated in neurons after oxidant damage [280]. Interestingly, upregulation of ROS levels in astrocytes induced by ischemic damage prompted mitochondrial transfer from bmMSCs to damaged astrocytes [279]. These findings could be useful for developing genetic approaches aimed at the modulation of mitochondrial trafficking genes to improve the efficacy of cell-based IS therapies. In addition, therapeutic hypothermia has been identified as a possible priming instrument able to induce Miro1 upregulation in MSCs. In fact, local cooling infusion (LCI) enhanced MSC mitochondrial transfer-mediated neuroprotection in IS, indicating that a combination of LCI and MSC transplantation may improve the clinical translation of cell-based IS therapies [281] (Figure 2).

In addition to MSCs, another promising candidate for IS cell-based therapies is endothelial progenitor cells (EPCs). EPCs, as MSCs, can be isolated from the bone marrow and are endowed with two advantages: They are able to circulate in the bloodstream, and they can take part in angiogenesis and vasculogenesis, two aspects that suggest their capability to migrate in and home to the CNS and to reconstitute the blood-brain barrier (BBB) [282,283]. In fact, in IS animal models, it has been shown that heterologous, homologous, and autologous EPCs are actively incorporated into angiogenetic sites [284]. To corroborate the fact that cellular angiogenetic abilities give increased therapeutic value to IS cell-based therapy, bmMSCs expressing exogenous angiopoietin-1 and VEGF genes showed greater structural–functional recovery in an MCAO rat model than normal bmMSCs [285]. Moreover, increasing EPC motility by parathyroid hormone (PTH) treatment enhanced CNS tissue repair and functional recovery in an MCAO mouse model [286]. Extracellular mitochondrial transfer via bmMSCs represents one of the mechanisms beyond EPC-driven BBB recovery. In vitro experiments showed that OGD increased transcellular endothelial permeability, with a consequent release of mitochondrial particles into the culture medium. Moreover, oxygen and glucose recovery induces a restoration of endothelial tightness, suggesting that EPCs may support both CNS energetics and angiogenic retrieval through mitochondrial transfer [282]. In addition, it has been found that circulating EPC levels represent a positive prognostic factor in patients affected by acute IS; indeed, they are increased in patients with smaller infarct volumes, and their reduction is associated with an increased risk of future vascular events [287,288,289]. Notably, patients affected by cerebrovascular diseases showed reduced levels of circulating EPCs, suggesting that they may have a broader role as prognostic factors in the pathophysiological disorders of the CNS [290].

## 4. Conclusions

To date, stroke therapy mainly relies on tissue plasminogen (tPA) administration and endovascular thrombectomy intervention for the acute phase, while physical therapy and cognitive rehabilitation are mainly applied in the long term [291,292,293]. Mitochondrial dysfunction is the main feature seen during the initiation of stroke pathophysiology; thus, targeting molecular pathways involved in IS pathology, such as mitochondrial ROS overproduction, mitochondrial Ca^2+^ overload, mPTP opening, inflammation, and aberrant mitochondrial dynamics, represents a promising strategy to attenuate cellular damage and cell death.

In recent years, extensive efforts have been made to discover and develop new drugs targeting mitochondrial dysfunction to efficiently rescue or ameliorate the outcomes of ischemic brain injury. Unfortunately, although pharmacological targeting of mitochondrial dysfunction has demonstrated a protective effect in preclinical studies, most of the neuroprotective drugs have showed limited benefits in clinical trials [294]. Therefore, an extensive characterization of mitochondrial protective mechanisms, taking into account the complex mechanisms by which each molecular player is connected to the others, may provide a rationale for the development of new therapeutic strategies. Among the mitochondrial protective mechanisms, mitochondrial transfer and transmitophagy, both aimed at the replenishment of healthy mitochondria in damaged cells, have come into the spotlight, and this increased understanding highlights a new perspective on the development of therapeutic approaches for IS. In fact, one of the modern trends of the neurological branch of regenerative medicine is the use of stem cells as surrogate sources of functional mitochondria for ischemia-threatened cells. To date, the majority of clinical trials have focused on the use of MSC transplantation as a therapeutic tool for IS (NCT01678534, NCT04280003, NCT04097652, NCT01922908, NCT02564328, NCT03384433, NCT04434768, NCT00875654, NCT02580019, NCT01716481, NCT01468064, NCT01297413, and NCT01714167). Only 4 clinical trials are based on the use of EPCs (NCT02157896, NCT01289795, NCT02605707, and NCT01468064), and only one aims to administer MSC-derived exosomes to stroke patients (NCT03384433). It is important to emphasize that despite the promising results observed, cell-based therapies could give rise to different adverse effects, such as embolization, infection, and immune-inflammatory responses, in addition to the difficulties in evaluating the correct number of functional cells that have to be transplanted to give effective results [281,283]. These limitations might be reasons for the limited clinical progress in IS cell-based therapies obtained in the past decades. In the future, fully understanding the molecular mechanisms underlying cellular mitochondrial transfer and MSC/EPC mobilization and migration to the ischemic site would allow the safe and effective development of functional IS therapies. Additionally, a complete characterization of mitochondrial involvement in IS to identify strategies to enhance their regenerative contribution is equally mandatory.

## Data Availability

Not applicable.

## Abbreviations

APAF-1	apoptotic protease activating factor 1
ATF4	activating transcription factor 4
bmMSCs	bone marrow derived-MSCs
CaMKIα	calcium/calmodulin-dependent protein kinase Iα
CDK1	cyclin dependent kinase 1
CL	cardiolipin
CNS	central nervous system
Cyt c	cytochrome c
DRP1	dynamin-related protein 1
EPCs	endothelial progenitor cells
ERK	extracellular signal-regulated-kinase
EVs	extracellular vesicles
FIS1	fission factor 1
FKBP8	FK506-binding protein 8
FUNCD1	FUN14 Domain Containing 1
i-AAA	intermembrane space ATPase
I/R	ischemic reperfusion
IL-18	interleukin-18
IL-1β	Interleukin-1β
IMM	inner mitochondrial membrane
IMS	mitochondrial intermembrane space
IS	ischemic stroke
L-OPA1	long optic atrophy 1
LCI	local cooling infusion
m-AAA	matrix ATPases
MAMs	mitochondria associated membranes
MCU	mitochondria calcium uniporter
MFF	mitochondrial fission factor
MFN1	mitofusin 1
MFN2	mitofusin 2
MID49	mitochondrial dynamics protein of 49 KDa
MID51	mitochondrial dynamics protein of 51 KDa
mPTP	1-methyl-4-phenyl-1,2,3,6-tetrahydropyridine
MSCs	mesenchymal stem cells
mtDNA	mitochondrial DNA
NBR1	neighbor of Brca1
NDP52	Nuclear Dot Protein 52
NIX/BNIP3L	NIP3-Like Protein X/BCL2 Interacting Protein 3 Like
NLRP3	nucleotide-binding domain and leucine-rich repeat containing protein 3
NMDA	N-methyl-D-aspartate
NRF-1	Nuclear Respiratory Factor 1
NRF-2	Nuclear Respiratory Factor 2
OGD	oxygen and glucose deprivation
OMA1	overlapping activity with m-AAA protease
OMM	outer mitochondrial membrane
OPA1	optic atrophy 1
OPTN	optineurin
OXPHOS	oxidative phosphorylation
p62/SQSTM1	p62/sequestosome 1
PARL	presenilin-associated rhomboid-like
PGC-1	peroxisome proliferator-activated receptor-γ coactivator 1 (known as PPAR-Gamma Coactivator 1)
PINK1	PTEN-induced kinase 1
PKA	cyclic AMP-dependent protein kinase
PKCδ	protein kinase C delta
ROCK1	Rho-associated coiled coil-containing protein kinase 1
ROS	reactive oxygen species
S-OPA1	short optic atrophy 1
SIRT1	Sirtuin 1
SOD2	superoxide dismutase 2
TAX1BP1	Tax1 Binding Protein 1
TBK1	TANK Binding Kinase 1
tMCAO	transient middle cerebral artery occlusion
TNFAIP2	TNF Alpha Induced Protein 2
TNTs	named tunneling nanotubes
tPA	tissue plasminogen
UCP2	uncoupling protein 2
Ψm	mitochondrial membrane potential
